# pNaKtide Inhibits Na/K-ATPase Signaling and Attenuates Obesity

**Published:** 2023-07-28

**Authors:** Komal Sodhi, Kyle Maxwell, Yanling Yan, Jiang Liu, Muhammad A Chaudhry, Zijian Xie, Joseph I Shapiro

**Affiliations:** Department of Medicine, Biomedical Science, and Surgery, Joan C. Edwards School of Medicine, Marshall University, Huntington, United States of America

**Keywords:** Obesity, Na/K-ATPase, Oxidative stress, Adipose tissue

## Abstract

Obesity is a growing public health crisis across the world and has been recognized as an underlying risk factor for metabolic syndrome. Growing evidence demonstrates the critical role of oxidative stress in the pathophysiological mechanisms of obesity and related metabolic dysfunction. As we have established previously that Na/K-ATPase can amplify oxidative stress signaling, we aimed to explore the effect of inhibition of this pathway on obesity phenotype using the peptide antagonist, pNaKtide. The experiments performed in murine preadipocytes showed the dose-dependent effect of pNaKtide in attenuating oxidant stress and lipid accumulation. Furthermore, these *in vitro* findings were confirmed in C57Bl6 mice fed a high-fat diet. Interestingly, pNaKtide could significantly reduce body weight, ameliorate systemic oxidative and inflammatory milieu and improve insulin sensitivity in obese mice. Hence the study demonstrates the therapeutic utility of pNaKtide as an inhibitor of Na/K-ATPase oxidant amplification signaling to alleviate obesity and associated comorbidities.

## INTRODUCTION

Obesity has become a worldwide epidemic with a significant increase in overweight population, giving rise to myriad of obesity associated comorbidities leading to increased mortality [1]. There has been increasing evidence for mechanistic link between oxidative stress in adipose tissue, consequently leading to the development and progression of obesity associated metabolic syndrome [2,3]. The progression of obesity associated comorbidities is not only stimulated by the systemic pro-oxidant milieu but can also be promoted by the altered redox state in adipocytes [4–6]. Furthermore, previous studies have extensively elucidated the potential of adipocyte-targeted anti-oxidative therapies, which have resulted in improved adipocyte phenotype, reduction in body weight and amelioration of metabolic abnormalities in murine models [7–10].

Chronic obesity attributes to altered adipogenesis which includes excessive lipid accumulation and impaired endocrine function of adipose tissue [11]. Several mediators have been identified that maintain adipogenic homeostasis, including Peroxisome Proliferator–Activated Receptor γ (PPARγ), which is known to be a master regulator of adipogenesis [12,13]. Studies have highlighted an important role of PPARγ in the activation of Fatty Acid Synthase (FAS), which stimulates fatty acid and triglyceride synthesis [14]. Mesoderm-Specific Transcript (MEST) is another notable marker of adipogenesis, upregulation of which has been shown to be associated with increased production of adipocytokines as well as promoting insulin resistance [15]. Since adipose tissue regulate energy metabolism through production of these adipocytokines, the pathophysiological consequences of dysregulated production of adipocytokines promotes chronic inflammation and exacerbates obesity associated risk factors such as hyperlipidemia, insulin resistance and other metabolic derangements [16–18].

Extensive investigations conducted in our laboratory have provided detailed insights into the role of Na^+^ and K^+^ dependent Adenosine Triphosphatase (Na/K-ATPase), a member of the P-type ATPase family. Traditionally, Na/K-ATPase has been recognized for its ion pumping function involving the intercellular transportation of Na^+^ and K^+^ ions through ATP hydrolysis. The functional Na/K-ATPase consists of two subunits, α and β, each with isoforms contributing uniquely to the overall Na/K-ATPase function [19,20]. Our previous investigations have elucidated the scaffolding role of the α1 subunit with Src kinase, leading to conformational changes in Na/K-ATPase that initiate downstream signaling cascades [21–23]. Notably, the “activation” of the Na/K-ATPase α1 subunit phosphorylates Src and modulates the Epithelial Growth Factor Receptor (EGFR), resulting in the generation of Reactive Oxygen Species (ROS) through the activation of pro-oxidant pathways [24,25]. In addition, our research group have demonstrated that the Na/K-ATPase α1 subunit can also function as a receptor for ROS. Given that Na/K-ATPase is both activated by and amplifies ROS, the Na/K-ATPase amplification loop plays a crucial role in exacerbating the pro-oxidant state under pathological conditions [26,27]. In this context, we have developed a cell-permeant peptide, pNaKtide, derived from the N domain of the α1 subunit, which specifically interacts with the kinase domain of Src, thereby antagonizing Na/K-ATPase signaling [28–31]. Considering the intricate association between oxidative stress and the development and progression of obesity and its related comorbidities, we hypothesize that pNaKtide may significantly ameliorate the obesity phenotype and associated adipogenesis, both *in vitro* and *in vivo*, by targeting the Na/K-ATPase amplification loop and attenuating ROS-mediated oxidative stress.

## MATERIALS AND METHODS

### Experimental design for *in vitro* experiment

3T3-L1 cells, obtained from ATCC, were reconstituted in α-Minimal Essential Medium (α-MEM) supplemented using 10% inactivated fetal bovine serum and 1% antibiotic/antimycotic solution. The cell cultures were maintained at 37°C in a 5% CO_2_ incubator, and the medium was replenished every 48 hours. Upon reaching 80% confluence, the cells were detached using trypsin. Subsequently, the cells were seeded in 96- and 24-well plates at a density 10,000 cells/cm^2^ and cultured until 80% confluence was achieved. Adipogenic medium was then added, and the cells were further cultured for 7 days. pNaKtide was administered daily at concentrations of 0.1 μM, 0.3 μM, 0.7 μM, 1 μM, and 2 μM. Adipogenesis was assessed after 7 days using Oil Red O staining [32]. Cell viability was determined using the MTT assay, as previously described [33]. To induce oxidative stress, 3T3-L1 cells were treated daily with glucose oxidase (3 mU/L) and fructose (500 μM), with and without pNaKtide (0.7 μM), for 7 days.

### Measurement of superoxide levels for *in vitro* experiment

3T3-L1 adipocytes were cultured on 96-well plates until reaching 70% confluence, followed by treatment with pNaKtide at concentrations of 0.3 μM, 0.7 μM, and 1 μM for duration of 72 hours. Subsequently, the cells were exposed to 10 mM dihydroethidium for 30 minutes at 37°C. The fluorescence intensity was measured using a Perkin-Elmer luminescence spectrometer with excitation/emission filters set at 530/620 nm [34].

### Experimental design for *in vivo* experiment

The *in vivo* studies were conducted following the guidelines outlined in the National Institutes of Health (NIH) Guide for the Care and Use of Laboratory Animals and approved by the Marshall University Animal Care and Use Committee. Male C57Bl6 mice (6–8 weeks old) were obtained from The Jackson Laboratory and housed at the Robert C. Byrd Biotechnology Science Center Animal Resource Facility (ARF). The mice were provided a standard chow diet and had ad libitum access to water. For the experimental groups, after being fed a high-fat diet for 4 weeks, the mice were divided into four groups and subjected to an 8-week treatment period. The high-fat diet, obtained from Bio-SERV, contained 58% fat (from lard), 25.6% carbohydrates, and 16.4% protein, yielding an energy density 23.4 kJ/g. The groups received intraperitoneal injections of pNaKtide, dissolved in saline, at doses of 1 mg/kg, 5 mg/kg, and 25 mg/kg every 8 days. Body weight was monitored weekly throughout the 12-week experimental period. At the end of the study, after an 8-hour fast, blood samples were collected from the tail vein for glucose measurement using a glucometer and insulin estimation using an Enzyme-Linked Immunosorbent Assay (ELISA) kit (Abcam Cambridge, MA). Body weight, visceral fat, and subcutaneous fat content were measured at the time of sacrifice. Plasma, obtained from the blood samples, was used to determine adiponectin levels. Visceral adipose tissue was rapidly frozen in liquid nitrogen and stored at −80°C until further analysis.

### Blood measurements of adiponectin

The measurement of high molecular weight adiponectin in plasma was performed using an Enzyme-Linked Immunosorbent Assay (ELISA) kit following the manufacturer’s protocol (Abcam Cambridge, MA).

### Western blot analysis

Following homogenization of visceral fat and 3T3-L1 adipocyte cell lysates in homogenization buffer, the resulting homogenates were subjected to centrifugation. The supernatant was collected and utilized for immunoblotting analysis of the following proteins: FAS, adiponectin, PPARγ, and MEST. β-Actin was employed as the loading control for all western blot experiments.

### Measurement of c-Src and ERK1/2 phosphorylation

The determination of c-Src and ERK1/2 was conducted following the established protocol described by Yan et al. using visceral fat and 3T3-L1 adipocyte cell lysates prepared with NP-40 buffer [26]. The membranes utilized for immunoblotting of phospho-c-Src and phospho-ERK1/2 was subsequently probed for total c-Src and total ERK1/2 after stripping. The ratios of phospho-c-Src/total Src and phospho-ERK1/2/total ERK1/2 were calculated after normalization with their respective control samples.

### Assessment of protein carbonylation

Protein carbonylation was quantified using visceral fat and whole-cell lysates prepared with NP-40 buffer, following the western blotting protocol described in previous studies [22,26]. Coomassie staining was employed as a loading control, and signal densities were normalized with control samples.

### Glucose tolerance test

Glucose clearance was assessed through an intraperitoneal glucose tolerance test performed prior to the termination of the experiment. Mice were fasted for 8 hours and subsequently injected with a glucose solution (2 g/kg, administered as a 10% solution) into the peritoneal cavity. Blood samples were collected from the tail vein at 0 minutes, 30 minutes, 60 minutes, and 120 minutes after glucose injection. Blood glucose levels were measured using the Accutrend Sensor glucometer.

### Determination of homeostasis model assessment of insulin resistance

The concentrations of glucose and insulin obtained following an 8-hour fasting period were utilized to calculate the Homeostasis Model Assessment of Insulin Resistance (HOMA-IR) using the formula: HOMA-IR=[fasting insulin (ng/mL) × fasting glucose (mM)]/22.5.

### Lipid peroxidation measurement

The estimation of Thiobarbituric Acid Reactive Substances (TBARS) was conducted using a commercially available assay kit (Cayman Chemical, Ann Arbor, MI) to quantify lipid peroxidation in visceral fat. Visceral fat tissue was homogenized in a buffer solution consisting of 50 mM tris-HCl (pH 7.4) and 1.15% KCl, followed by centrifugation to obtain the supernatant for the assay. The obtained values were normalized to the corresponding total protein content and expressed as micromoles per milligram of protein.

### Distribution of rhodamine B–labeled pNaKtide

The cellular distribution of rhodamine B-labeled pNaKtide in a cell culture model was evaluated using murine preadipocytes. 3T3-L1 cells were cultured on glass coverslips and treated with or without rhodamine B-labeled pNaKtide (2 μM) for specific time periods. Subsequently, the cells were fixed using cold absolute methanol and mounted with 4’,6-Diamidino-2-Phenylindole (DAPI) mounting medium (Vector Labs). Emission readings for DAPI and rhodamine B were recorded at 415 nm to 475 nm and 580 nm to 650 nm, respectively. Imaging was performed using a Leica SP5 TCS II confocal microscope equipped with a coherent chameleon multiphoton vision II (IR) laser, and image analysis was carried out using Leica LAS/AF software. In the *in vivo* system, mice were intraperitoneally injected with rhodamine B-labeled pNaKtide (25 mg/kg) or without (as control) to investigate the distribution of pNaKtide. The mice were sacrificed at specific time intervals, and adipose tissues were imaged and analyzed following the same procedure as described above.

### Statistical analysis

The results are plotted using box-and-whisker plots, with each plot representing the maximum and minimum range, upper quartile, median and lower quartile values. All statistical analysis was performed using GraphPad Prism version 9 (GraphPad, San Diego, CA, USA). Statistical significance was assigned at p<0.05 or p<0.01 for confidence interval of 95% or 99%, respectively. One-way Analysis of Variance (ANOVA) was used for comparing multiple groups followed by Tukey’s post hoc test, while an unpaired two-tailed t-test was employed for comparing two groups, both aimed at testing the null hypothesis. The group comparison employed for each results and the sample size are detailed in the respective figure legends.

## RESULTS

### Effect of pNaKtide treatment on adipogenesis in murine adipocytes

**Figure 1:**
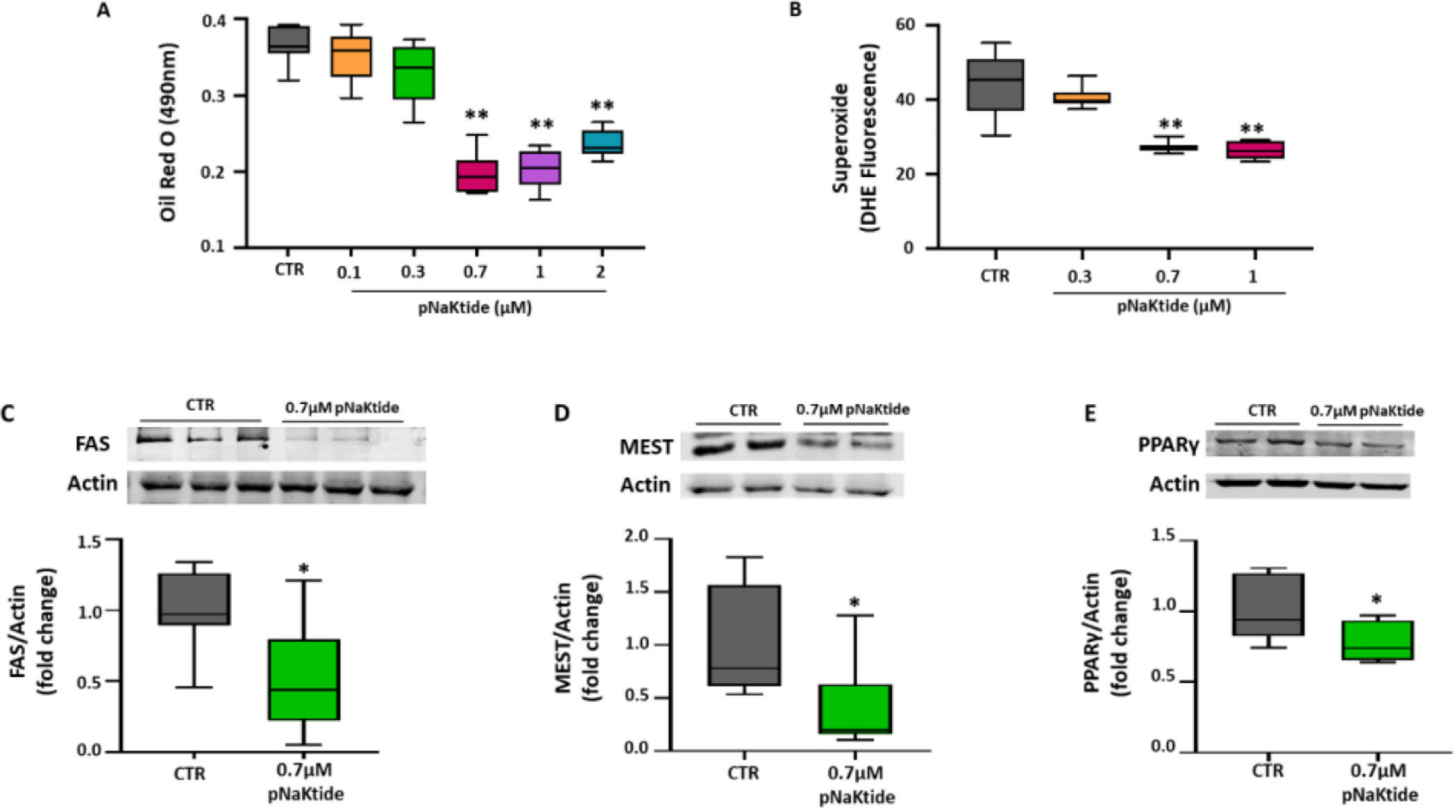
pNaKtide improves adipogenesis in murine adipocytes. To evaluate the effect of pNaKtide on adipogenesis and determine optimal concentration, murine preadipocytes were cultured with varying concentrations of pNaKtide. (A) Quantitative assessment of relative absorbance by Oil Red O staining to measure adipogenesis in murine preadipocytes cultured in adipogenic media with varying concentrations of pNaKtide, (n=7/group); (B) Dihydroethidium (DHE) staining to measure superoxide levels, (n=6/group); Western blot analysis in murine adipocyte treated with 0.7 μM of pNaKtide for protein expression of adipogenic markers, (C) FAS (n=8/group), (D) MEST (n=8/group) and (E) PPARγ (n=7/group), with mean band density normalized to actin. **Note:** Each box and whisker plot represents values as maximum and minimum range, upper quartile, median and lower quartile. *p<0.05 *vs.* CTR, **p<0.01 *vs.* CTR.

Murine preadipocytes, 3T3L1 cells, were cultured in adipogenic medium, to induce adipogenesis, and exposed to varying concentration of pNaKtide to determine optimum concentration. Our results showed significant reduction of lipid accumulation in 3T3L1 adipocytes treated with all increasing concentrations of pNaKtide from 0.7 μM to 2 μM, as measured by relative absorbance of Oil Red O (Figure 1A). To serve as a control, these 3T3L1 adipocytes were also cultured with rhodamine-labeled pNaKtide to illustrate the cellular distribution of pNaKtide at varying time-intervals. Our results showed that pNaKtide readily crossed the cell membrane of 3T3L1 adipocytes with maximum distribution in the compartments of intracellular membrane at 2 hours (Figure S1A). Since activation of Na/K-ATPase signaling causes increase in oxidative stress, we aimed to determine whether pNaKtide, by antagonism of Na/K-ATPase signaling, can attenuate oxidative stress in 3T3L1 adipocytes. Our results showed significant reduction in the levels of superoxide, a marker of oxidative stress, by treatment at 0.7 μM and 1.0 μM of pNaKtide in 3T3L1 adipocytes (Figure 1B). No cytotoxic effects of pNaKtide were noted at the studied doses (up to 2 μM) as evidenced by MTT assay. Briefly, the results of MTT assay showed no significant changes among groups (*viz* mean values as Control=2.72 ± 0.11; 0.1 μM pNaKtide=2.69 ± 0.14; 0.3 μM pNaKtide=2.73 ± 0.09; 0.7 μM pNaKtide=2.94 ± 0.12; 1 μM pNaKtide=3.05 ± 0.15; 2 μM pNaKtide=2.96 ± 0.16).

Based on these observations, 0.7 μM of pNaKtide was deemed to be the optimal concentration to induce beneficial effect in 3T3L1 adipocytes. Subsequently, our results further showed significant downregulation in the expression of adipogenic markers, FAS, MEST and PPARγ, in 3T3L1 adipocytes treated with 0.7 μM of pNaKtide (Figures 1C,1D, and 1E). Collectively, our results demonstrated that pNaKtide was effective in reverting adipocyte dysfunction, caused by the Na/K-ATPase signaling mediated oxidative stress.

### Effect of pNaKtide on body weight and adipose tissue mass in C57BL6 mice fed a high fat diet

**Figure 2:**
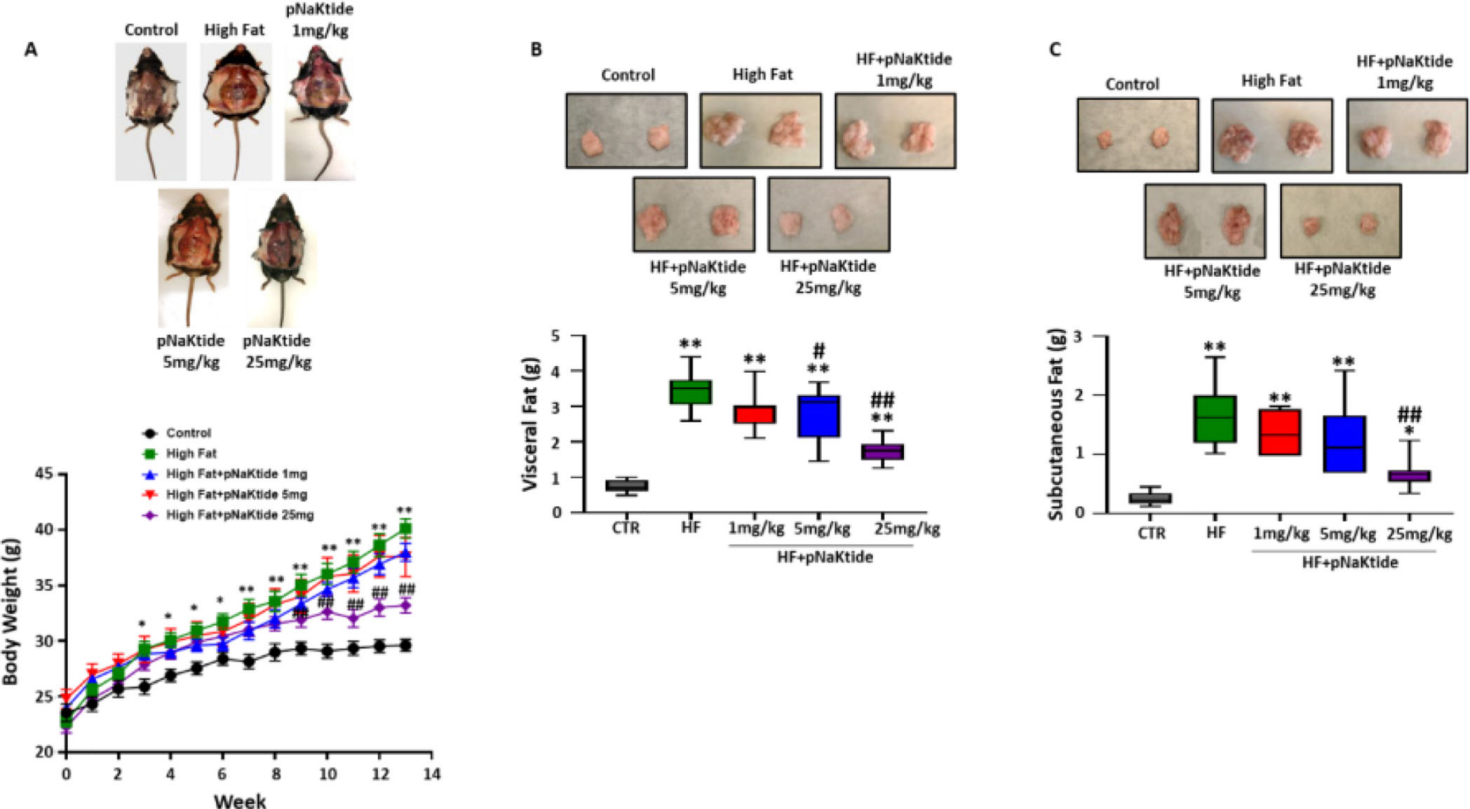
pNaKtide improves body weight and adipose tissue mass in dose-dependent manner in high fat diet-fed mice. Mice were fed either normal chow or high fat diet with or without varying concentrations of pNaKtide, including 1 mg/kg, 5 mg/kg and 25 mg/kg. (A) Body weight of mice measured over 12 weeks of study period (n=7–21/group); Line plot representing mean values, with their standard errors represented by vertical bars. *p<0.05 and **p<0.01 CTR *vs*. HF, ##p<0.01 HF *vs*. HF+pNaKtide 25 mg (B) Visceral adipose tissue mass (n = 7–20/group) and (C) Subcutaneous adipose tissue mass (n=7–20/group), in mice fed a high fat diet with or without varying concentration of pNaKtide. **Note:** Each box and whisker plot represents values as maximum and minimum range, upper quartile, median and lower quartile. *p<0.05 *vs*. CTR, **p<0.01 *vs*. CTR, #p<0.05 *vs*. HF, ##p<0.01 *vs*. HF.

In one subset of study, C57BL6 mice were administered with rhodamine-labeled pNaKtide to show that pNaKtide accumulate in adipose tissue. Our results showed most efficient distribution of rhodamine-labeled pNaKtide in adipose tissues after 12 hours, when administered intraperitoneally in mice (Figure S1B). To demonstrate the effect of pNaKtide in improving obesity and to determine optimal dose for murine model, C57BL6 mice were fed a high fat diet for 4 weeks, followed by intraperitoneal administration of varying doses of pNaKtide (1 mg/kg, 5 mg/kg and 25 mg/kg of body weight, respectively) every 8 days, and high fat diet continued for another 8 weeks. Our results showed significant reduction in body weight of high-fat diet fed mice by intraperitoneal administration of pNaKtide at 25 mg/kg, while pNaKtide at 1 mg/kg and 5 mg/kg demonstrated less effect on the body weight over the course of 8 weeks (Figure 2A). Subsequently, our results showed significant reduction in visceral adipose tissue mass of mice administered with pNaKtide at 5 mg/kg, with even further reduction noted in mice administered with pNaKtide at 25 mg/kg, as compared to mice fed a high fat diet alone (Figure 2B). Furthermore, our results showed significant reduction in subcutaneous adipose tissue mass only in mice administered with pNaKtide at 25 mg/kg, as compared to mice fed a high fat diet alone (Figure 2C).

### Effect of pNaKtide on adipogenesis and adiponectin expression in C57BL6 mice fed a high fat diet

**Figure 3:**
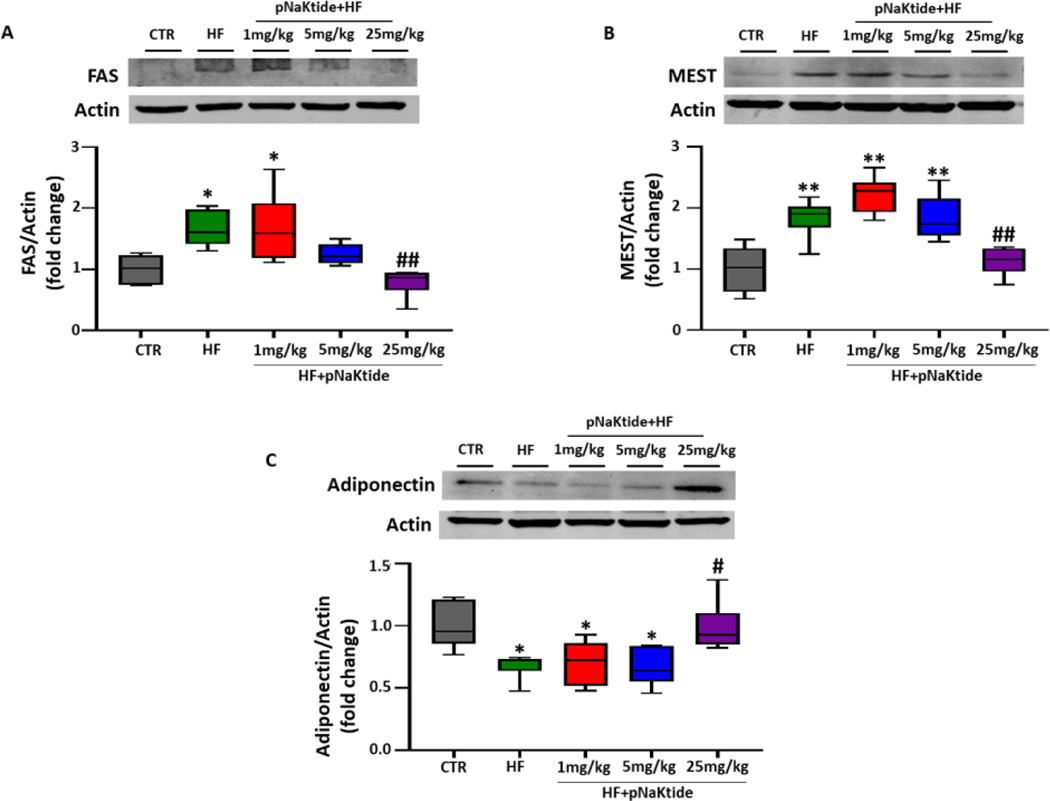
pNaKtide improves adipogenesis and adiponectin expression in dose-dependent manner in high fat diet-fed mice. Western blot analysis in mice fed a high diet with or without varying concentration of pNaKtide for protein expression of (A) FAS (n=6/group), (B) MEST (n=6/group) and (C) adiponectin (n=6/group), with mean band density normalized to actin. **Note:** Each box and whisker plot represents values as maximum and minimum range, upper quartile, median and lower quartile. *p<0.05 *vs*. CTR, **p<0.01 *vs*. CTR, #p<0.05 *vs*. HF, ##p<0.01 *vs*. HF.

Our results showed a significant increase in the expression of adipogenic markers, FAS and MEST, in the visceral adipose tissues of mice fed a high fat diet, as compared to control (Figures 3A and 3B). Administration of pNaKtide at 25 mg/kg in high fat diet fed mice significantly attenuated the expression of these adipogenic markers, as compared to mice fed a high fat diet (Figures 3A and 3B). Similarly, high fat diet fed mice administered with pNaKtide at 25 mg/kg showed significant upregulation of adiponectin expression, as compared to high fat diet alone, suggestive of the role of pNaKtide in promoting development of healthy adipocytes (Figure 3C).

### Effect of pNaKtide on metabolic profile, oxidative stress, and inhibition of the Na/K-ATPase signaling cascade in C57BL6 mice fed a high-fat diet

**Figure 4:**
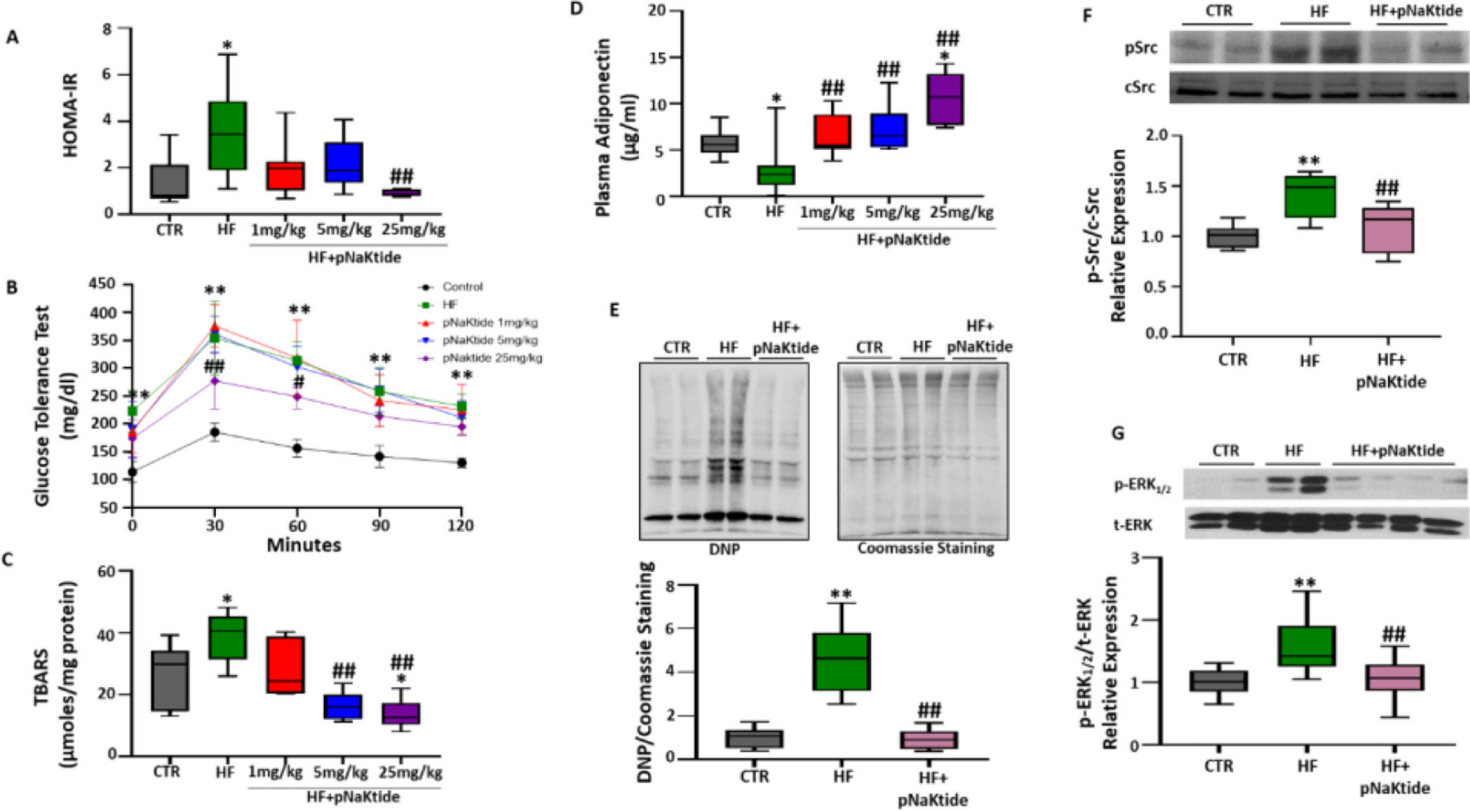
pNaKtide improves metabolic profile, oxidative stress and inhibits Na/K-ATPase signaling cascade in mice fed a high fat diet. Dose- dependent effect of pNaKtide was determined by (A) Estimation of insulin resistance by HOMA-IR score, (n=6–7/group); (B) Blood glucose levels measured in glucose tolerance test, (n=6–8/group). Line plot representing mean values, with their standard errors represented by vertical bars. **p<0.01 CTR *vs*. HF, #p<0.05 and ##p<0.01 HF *vs*. HF+pNaKtide 25 mg; (C) Quantitative assessment of TBARS in visceral adipose tissue, (n=7/group). (D) Plasma adiponectin levels measured by ELISA, (n=7–19/group); (E) Quantitative assessment of protein carbonylation levels shown as DNP expression, normalized with Coomassie loading control, (n=10/group); Immunoblot analysis of (F) pSrc, with mean band density normalized to total Src, (n=8/group) and (G) pERK1/2 with mean band density normalized to total ERK, (n=6–12/group) to demonstrate antagonism of Na/K-ATPase signaling and inhibition of downstream mediators by pNaKtide. **Note:** Each box and whisker plot represents values as maximum and minimum range, upper quartile, median and lower quartile. *p<0.05 *vs*. CTR, **p<0.01 *vs*. CTR, #p<0.05 *vs*. HF, ##p<0.01 *vs*. HF.

Our results demonstrated that administration of pNaKtide at concentration of 25 mg/kg was effective in preventing insulin resistance in high fat diet fed mice (Figure 4A). This was further corroborated by glucose tolerance test as our results showed significant improvement of glucose tolerance in high fat diet fed mice administered with pNaKtide, as compared to mice only fed with high fat diet (Figure 4B). Administration of pNaKtide also resulted in marked reduction of oxidative stress in the visceral adipose tissues of high fat diet fed mice, as determined by the TBARS assay (Figure 4C). Furthermore, assessment of plasma adiponectin showed significantly increased levels in high fat diet fed mice administered with pNaKtide (Figure 4D). Subsequently, protein carbonylation assessed by expression of DNP, a marker of oxidative stress, was also downregulated by the administration of pNaKtide in high fat diet fed mice, as compared to mice fed only high fat diet (Figure 4E). Since high fat diet mediated carbonylation of Na/K-ATPase α1 subunit initiates a signaling cascade and Phosphorylates Src (pSrc) kinase and downstream ERK1/2 (pERK1/2), our results confirmed antagonism of Na/K-ATPase signaling, as noted by significant attenuation of pSrc and downstream pERK1/2 expression by pNaKtide in high fat diet fed mice (Figures 4F and 4G).

### Effect of pNaKtide on adipogenesis in glucose oxidase treated murine adipocytes

**Figure 5:**
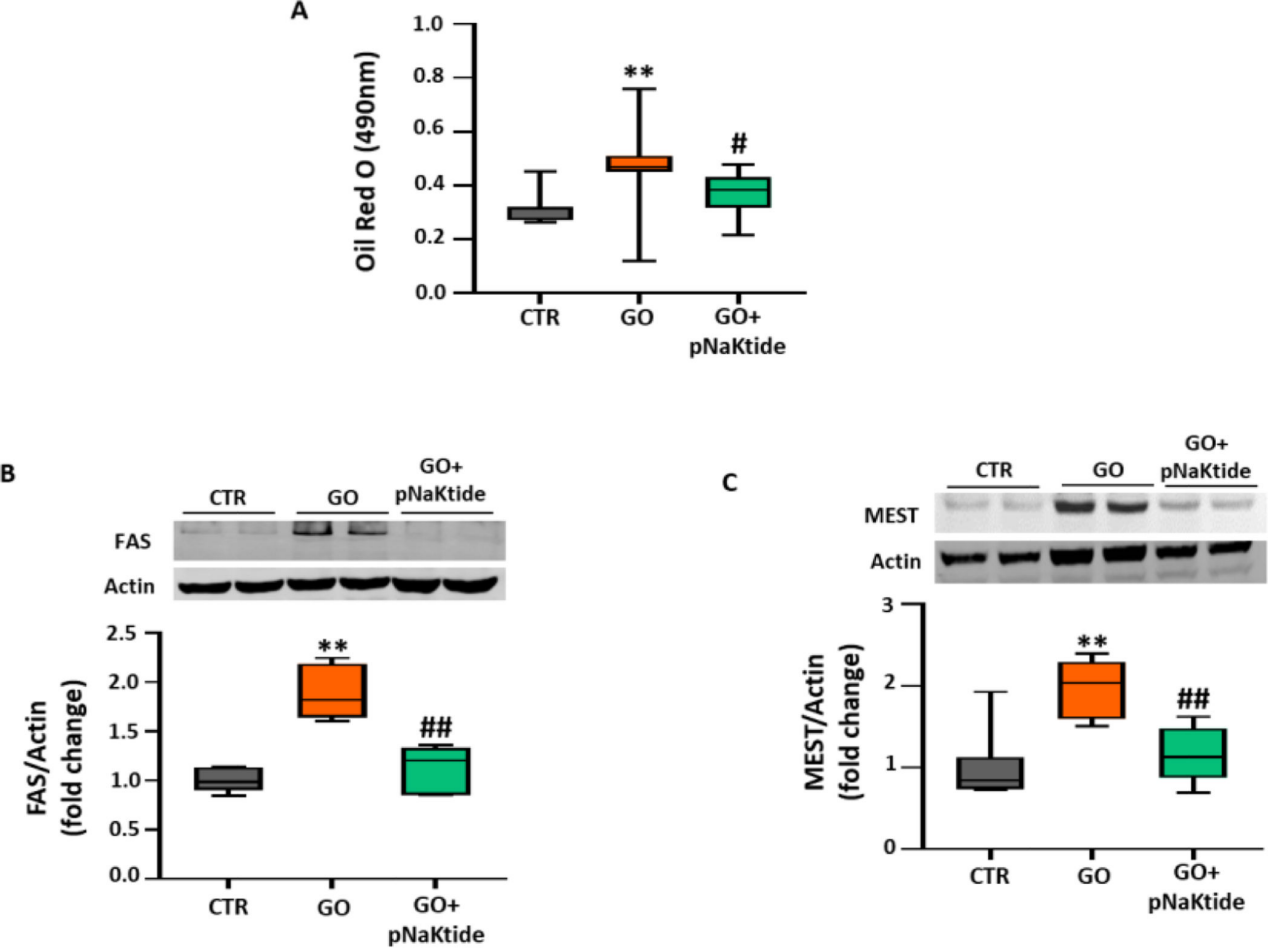
pNaKtide improves adipogenesis, oxidative stress and inhibits Na/K-ATPase signaling cascade in murine adipocytes exposed to glucose oxidase. Murine preadipocytes were cultured in adipogenic medium and exposed to glucose oxidase with or without 0.7 μM of pNaKtide. (A) Quantitative assessment of relative absorbance by Oil Red O staining to measure adipogenesis, (n=8–16/group); Western blot analysis for protein expression of adipogenic markers, (B) FAS (n=6/group) and (C) MEST (n=7/group), with mean band density normalized to actin. **Note:** Each box and whisker plot represents values as maximum and minimum range, upper quartile, median and lower quartile. *p<0.05 *vs*. CTR, **p<0.01 *vs*. CTR, #p<0.05 *vs*. GO, ##p<0.01 *vs*. GO.

Studies have shown that glucose oxidase, induce oxidative stress and exacerbate lipid accumulation, when exposed to 3T3L1 cells [35,36]. Hence, to further corroborate our findings that pNaKtide attenuates oxidative stress and inhibits adipogenesis, 3T3L1 cells cultured in adipogenic medium were exposed to glucose oxidase with or without 0.7 μM of pNaKtide. Our results showed that the increase in lipid accumulation by glucose oxidase, as compared to control, was significantly attenuated by treatment with pNaKtide (Figure 5A). In concordance, our results showed significant upregulation in the expression of adipogenic markers, FAS and MEST, in glucose oxidase treated 3T3L1 cells cultured in adipogenic medium (Figures 5B and 5C). Treatment with pNaKtide significantly attenuated the expression of these adipogenic markers (Figures 5B and 5C). Hence, these findings suggest that antagonism of Na/K-ATPase signaling by pNaKtide improves glucose oxidase mediated exacerbation of adipogenesis.

### Effect of pNaKtide on adipogenesis in fructose treated murine adipocytes

**Figure 6:**
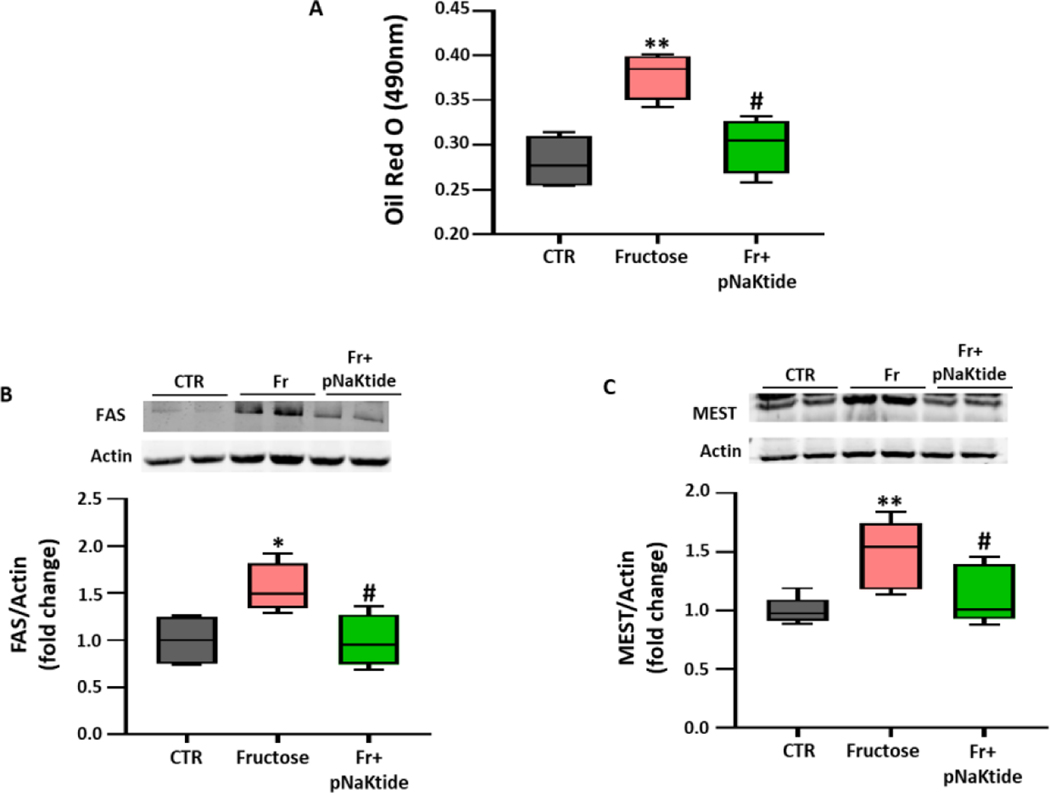
pNaKtide improves adipogenesis in murine adipocytes exposed to fructose. Murine preadipocytes were cultured in adipogenic medium and exposed to fructose (500 μM concentration) with or without 0.7 μM of pNaKtide. (A) Quantitative assessment of relative absorbance by Oil Red O staining to measure adipogenesis, (n=4/group); Western blot analysis for protein expression of adipogenic markers, (B) FAS (n=4/group) and (C) MEST (n=6/group), with mean band density normalized to actin. **Note:** Each box and whisker plot represent values as maximum and minimum range, upper quartile, median and lower quartile. *p<0.05 *vs*. CTR, **p<0.01 *vs*. CTR, #p<0.05 *vs*. Fr, ##p<0.01 *vs*. Fr.

Next, we aimed to determine whether fructose mediated increase of adipogenesis, through induction of oxidative stress, can be attenuated by pNaKtide treatment in 3T3L1 adipocytes. Our results showed significantly increased lipid accumulation in fructose treated 3T3L1 adipocytes, which was significantly attenuated by pNaKtide treatment (Figure 6A). Our results also showed significant upregulation in the expression of adipogenic markers, FAS and MEST, in fructose treated 3T3L1 adipocytes (Figures 6B and 6C). pNaKtide treatment attenuated the expression of these adipogenic markers (Figures 6B and 6C).

In Figures 1–6: Line plot representing mean values, with their standard errors represented by vertical bars; Each box and whisker plot represents values as maximum and minimum range, upper quartile, median and lower quartile.

## DISCUSSION

The present study demonstrates the implications of adipose tissue oxidative stress and associated metabolic dysregulations in the pathophysiology of obesity. Adiposity associated oxidative stress is an instigator of metabolic syndrome and thus the redox state in adipose tissue is a potentially useful therapeutic target for obesity associated metabolic syndrome [37]. Antioxidants have gained attention because of their capacity to counteract the deleterious effects of free radicals and pathologies associated with them [38,39]. Hence, here in this study, we have demonstrated that antagonism of Na/K-ATPase–mediated redox signaling, by pNaKtide, effectively normalizes adiposity and restores metabolic homeostasis.

The manifestations of obesity phenotype in murine preadipocytes, in response to exogenous administration of oxidative stress mediators, have been significantly ameliorated by pNaKtide administration. pNaKtide significantly attenuated oxidative stress and excess lipid accumulation, in response to fructose and glucose oxidase, in a dose dependent manner. We observed a significant down-regulation in the expression of genes involved in adipogenic regulation including PPARγ, MEST and FAS in pNaKtide treated murine preadipocytes. These findings demonstrate that blockage of Na/K-ATPase signaling by pNaKtide that effectively prevents dysfunctional adipogenesis, which could be due to its effect on cellular redox. Further we confirmed these observations in *in vivo* model of obesity induced by a high-fat diet. Results showed that intraperitoneal administration of pNaKtide significantly attenuated the development of obesity and associated metabolic dysfunction. We observed a significant decrease in weight gain along with the attenuation of visceral and subcutaneous fat content in mice fed a high-fat diet treated with pNaKtide. The pathophysiology of obesity-induced metabolic diseases has been attributed to adipocyte dysfunction associated with oxidative stress, insulin resistance and hyperglycemia that further trigger the activation of pro-inflammatory signaling pathways [40]. There was a significant reduction in oxidative stress as evidenced by decreased TBARS and protein carbonylation in high fat mice treated with pNaKtide. In addition, pNaKtide decreased the insulin resistance along with an increase in adiponectin levels. Our results further demonstrated that the decrease in fat content was associated with alteration in adipogenic proteins. There was a significant down regulation of the expression of FAS and MEST in pNaKtide treated high-fat mice, further demonstrating the pNaKtide-mediated modulation of adiposity and metabolic dysfunction.

## CONCLUSION

To summarize, our results clearly demonstrate that Na/K-ATPase can exacerbate oxidative stress signaling associated with adipogenesis, which is not yet elucidated in relation with cardiotonic steroids or the Na/K-ATPase signaling. The study explores the pharmacological utility of a novel, anti-obesity drug that can be a potent candidate for future studies. Of note, the study provides new approaches for the treatment of obesity and associated metabolic syndrome. Furthermore, Na/K-ATPase mediated ROS amplification loop can be a potential therapeutic target in various pathophysiological conditions characterized by oxidative stress. Future studies involving human clinical trials are warranted to explore the clinical utility of pNaKtide for therapeutic applications. We believe that mechanisms mediated by this oxidant amplification loop in adipocytes might serve as a therapeutic target in obesity and associated metabolic alterations. We further suggest that therapies developed based on the targeting of this Na/K-ATPase signaling systemically or confined to adipocytes might ultimately have application in finding alternate pathways to develop new and effective therapies to limit the impact these conditions will have on the general population.

## Supplementary Material

Supplementary file_J Clin Med, Vol. 7 Iss. 4 No: 1000238
