# Supplementary material for: pNaKtide Inhibits Na/K-ATPase Signaling and Attenuates Obesity
Source: J Clin Med Sci. Author manuscript; Available in PMC 2024 Jan 26. (PMC10812088)
Supplement: Supplementary file_J Clin Med, Vol. 7 Iss. 4 No: 1000238 [file NIHMS1917373-supplement-Supplementary_file_J_Clin_Med__Vol__7_Iss__4_No__1000238.pdf]

ISSN: 2593-9947
Journal of Clinical and Medical Sciences

OPEN ACCESS Freely available online

Research Article

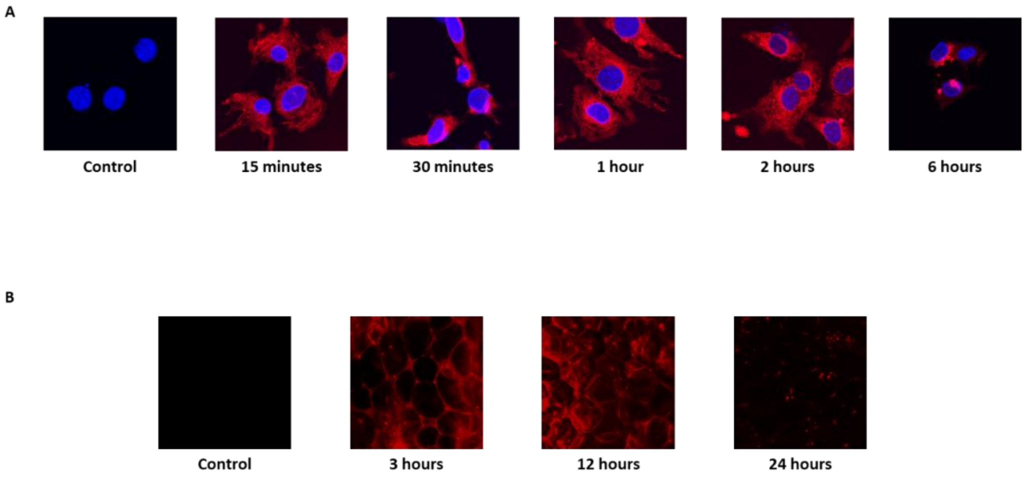

Figure 1: A: Distribution of rhodamine-labeled pNaKtide in murine preadipocytes. Murine preadipocytes were cultured with rhodamine-labeled-pNaKtide to assess cellular distribution at different time intervals. Representative images demonstrate maximum distribution of pNaKtide at 2-hour interval; B: Distribution of rhodamine-labeled pNaKtide by intraperitoneal injection in adipose tissue of mice. Mice were intraperitoneally injected with rhodamine-labeled-pNaKtide to assess distribution of pNaKtide in adipose tissue at different time intervals.

Correspondence to: Komal Sodhi, Department of Medicine, Biomedical Science, and Surgery, Joan C Edwards School of Medicine, Marshall University, Huntington, United States of America, E-mail: sodhi@marshall.edu

Received: 27-Jun-2023, Manuscript No. JCMS-23-21989; **Editor assigned:** 30-Jun-2023, Pre QC No. JCMS-23-21989 (PQ); **Reviewed:** 14-Jul-2023, QC No. JCMS-23-21989; **Revised:** 21-Jul-2023, Manuscript No. JCMS-23-21989 (R); **Published:** 28-Jul-2023, DOI: 10.35248/2593-9947.23.7.238

Citation: Sodhi K, Maxwell K, Yan Y, Liu J, Chaudhry MA, Xie J, et al. (2023) pNaKtide Inhibits Na/K-ATPase Signaling and Attenuates. J Clin Med Sci. 23:238.

Copyright: © 2023 Sodhi K, et al. This is an open access article distributed under the terms of the Creative Commons Attribution License, which permits unrestricted use, distribution, and reproduction in any medium, provided the original author and source are credited.
